# Effects of A Magnetic Field on the Transport and Noise Properties of a Graphene Ribbon with Antidots

**DOI:** 10.3390/nano10112098

**Published:** 2020-10-23

**Authors:** Paolo Marconcini, Massimo Macucci

**Affiliations:** Dipartimento di Ingegneria dell’Informazione, Università di Pisa, Via G. Caruso 16, I-56122 Pisa, Italy; m.macucci@mercurio.iet.unipi.it

**Keywords:** graphene ribbon, antidot, magnetic field, conductance, shot noise

## Abstract

We perform a numerical simulation of the effects of an orthogonal magnetic field on charge transport and shot noise in an armchair graphene ribbon with a lattice of antidots. This study relies on our envelope-function based code, in which the presence of antidots is simulated through a nonzero mass term and the magnetic field is introduced with a proper choice of gauge for the vector potential. We observe that by increasing the magnetic field, the energy gap present with no magnetic field progressively disappears, together with features related to commensurability and quantum effects. In particular, we focus on the behavior for high values of the magnetic field: we notice that when it is sufficiently large, the effect of the antidots vanishes and shot noise disappears, as a consequence of the formation of edge states crawling along the boundaries of the structure without experiencing any interaction with the antidots.

## 1. Introduction

Graphene, a planar hexagonal lattice of carbon atoms, represents one of the currently most studied and promising materials [1,2,3,4,5]. The interest raised by graphene has more recently also triggered large scale research on a wide range of 2D materials [6,7,8]. Graphene has very useful characteristics [9,10], such as high mechanical strength, electrical and thermal conductivity, flexibility and transparency.

Since its envelope-function transport equation is formally equivalent to the relativistic Dirac equation [11,12,13,14], graphene exhibits relativistic effects at velocities much smaller than the velocity of light, such as Klein tunneling and Zitterbewegung [15,16,17,18]. Moreover, it is characterized by peculiar electrical noise characteristics [19,20,21,22]. Furthermore, its behavior in the presence of high magnetic fields is particularly interesting [1,23,24,25], since it exhibits an “anomalous” quantum Hall effect, observable even at room temperature, with a spectrum of unevenly spaced Landau levels and a Landau level at zero energy.

Due, in particular, to its very high mobility and its current carrying capability, graphene has been often considered as a candidate to replace commonly used semiconductors, such as silicon, in “Beyond Moore” nanoelectronics [4,26,27,28,29,30,31,32]. Unfortunately, however, pristine graphene does not have an energy gap, which makes it difficult to effectively “turn off” a graphene-based transistor, and makes it unsuitable for digital electronics, for which a minimum $I_{on}/I_{off}$ ratio (the ratio of the current when the device is on to that when the device is off) of at least $10^{4}$ is required (while with pristine graphene it is possible to obtain at most a value of about 10).

It has been shown that a gap can be opened in graphene by means of lateral confinement, i.e., by defining a nanoribbon [14,33,34]; however, in order to achieve a gap of practical interest of a few hundreds of millielectronvolts, nanoribbons with a width of just a few nanometers should be defined, which is substantially impossible with current lithographic techniques. Chemical synthesis can yield such nanoribbons [35], but they are produced in a solution, and there is no practical approach for transferring millions or billions of them reliably and cost-effectively onto a substrate, in order to create circuits. Even the most recent advances [36] in nanoribbon direct synthesis on a metal oxide surface do not offer the possibility of placing the nanoribbons according to a predefined scheme.

Other methods have been proposed for opening up an energy gap in graphene [4,37], and, in particular, the introduction (by means of techniques such as e-beam lithography, diblock copolymer, nanosphere and nanoimprint lithography [38,39,40,41,42,43,44]) of a lattice of perforations (antidots) [45,46,47].

**Figure 1:**
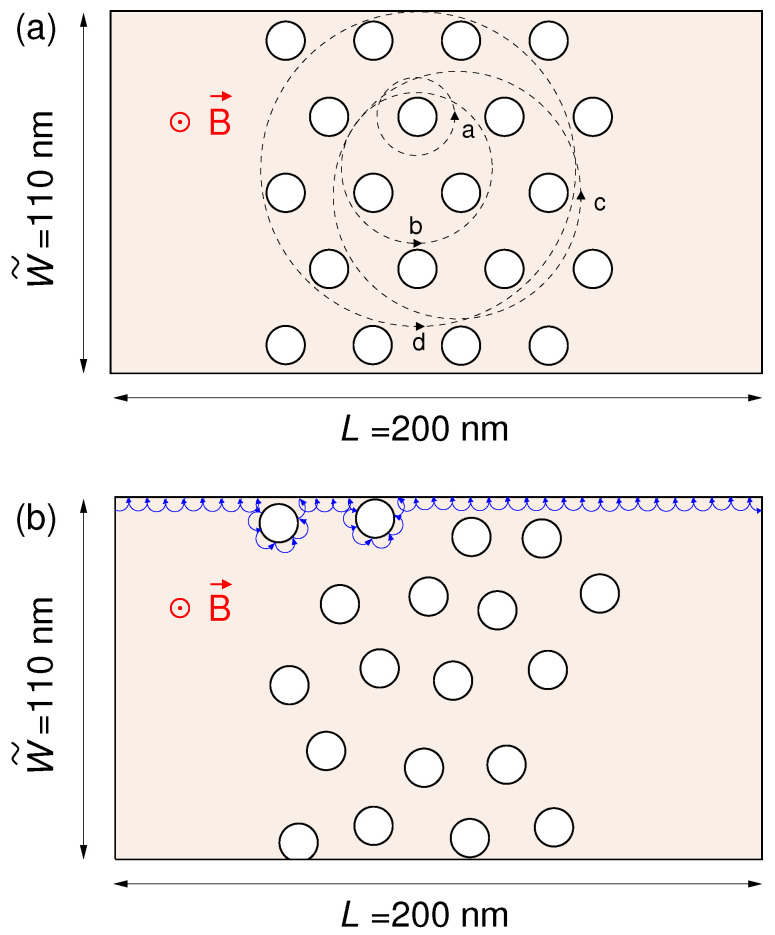
Considered graphene ribbon with a regular (**a**) and an irregular (**b**) distribution of circular antidots.

We performed a numerical investigation of the effects of an orthogonal magnetic field *B* on the transport and noise behavior of armchair graphene ribbons both with a regular and an irregular distribution of antidots (Figure 1).

The effects of the magnetic field on the transport properties of graphene samples with an antidot lattice have been experimentally explored in references [41,48,49,50]. Previous numerical studies on antidot lattices in graphene, performed using a tight-binding technique in references [51,52,53], have focused on the band-gap quenching in unconfined graphene and on conductance oscillations, related to the presence of edge states and of commensurability effects. Other analyses have instead focused on the effects of single antidots on transport in graphene in the presence of a magnetic field [54,55,56].

We have generalized our in-house developed envelope-function based transport simulator [57,58,59,60,61]: this code can efficiently handle relatively large graphene structures, with sizes of the order of hundreds of nanometers or microns (similar to those of the samples on which experimental studies are performed), which cannot be studied using more detailed but computationally demanding atomistic approaches. We note that the energy gap that is present for B=0 gradually vanishes as the applied magnetic field is increased. This represents a generalization of the conclusions of references [62] and [51]. In these references, an atomistic (tight-binding) approach was used to investigate the dependence of the energy gap on the magnetic field in graphene structures with very small feature sizes. In particular, in reference [62], narrow, unperforated (i.e., without antidots) graphene ribbons were analyzed, investigating the reduction of the energy gap with increasing magnetic field strength: as the highest valence band and the lowest conduction band get closer to each other [62], they ultimately coalesce for high values of the magnetic field strength, giving rise to the zero-energy Landau level characteristic of graphene. Instead, in reference [51], an unconfined, perforated (i.e., with antidots) graphene sheet with small feature sizes was studied; it was shown that the energy gap introduced by the antidot lattice should progressively disappear as the magnetic length approaches the lattice feature size. Our simulations extend these results to the case in which confinement and antidot lattice are simultaneously present, using a continuum model, which can handle relatively large graphene structures.

We also confirm the appearance of the commensurability effects and of the quantum oscillations, which have been previously experimentally reported and simulated [48,49,50,52].

Finally, we focus on the behavior for high values of the magnetic field: in this condition, the formation of edge states with small cyclotron radius, crawling along the boundaries of the ribbon without experiencing any scattering from the antidots, leads to a transport behavior in which the role of the antidots effectively disappears and shot noise vanishes. Similar phenomena have been reported in the two-dimensional electron gas of semiconductor heterostructures [63,64], where the antidots consisted simply in the electrostatic effect of charged impurities and defects. In graphene, the presence of Klein tunneling [15,16,17,18] and thus the impossibility to confine charge carriers only by means of the electrostatic potential landscape, make it necessary to implement the antidots by means of actual perforations in the graphene sample.

## 2. Method

We have added to our code for the k·p (envelope-function) [14] simulation of armchair graphene ribbons the capability of treating the effect of antidots and of an orthogonal magnetic field. In order to preserve the possibility to handle relatively large devices, we decided to neglect the atomistic details of the antidot edges by simulating the effect of the antidots with the introduction into the graphene envelope-function equation (Dirac equation) of a mass term $mv_{F}^{2}$ (where *m* is the mass and $v_{F}$ is the graphene Fermi velocity) [17,19,54,65], with a value that outside the antidots is zero and inside the antidots is much greater than the maximum energy of the moving charges. Regarding the magnetic field $\vec{B}=B\hat{z}$ (orthogonal to the plane x,y of the ribbon, where *x* and *y* are the transport and transverse directions, respectively), its effect on transport was simulated by introducing a vector potential $\vec{A}\left(x,y\right)$, such that $\vec{B}=\vec{\nabla }\times \vec{A}$, in the Dirac equation. In particular, among the infinite forms of vector potential $\vec{A}$ corresponding to the magnetic field $\vec{B}=B\hat{z}$, in most of our simulations we used the Landau gauge $\vec{A}=Bx\hat{y}$ [66].

We compute the transmission matrix of the device using a recursive scattering matrix technique [57]. We subdivide the structure into a series of sections, in each of which the potential energy U(x,y), the vector potential $\vec{A}\left(x,y\right)$ and the mass term m(x,y) are approximately longitudinally constant. In particular, with our choice of gauge, where $\vec{A}$ depends on *x*, the condition on the longitudinal invariance of the vector potential within each section translates into a limitation on the magnetic flux threading each section, which has to be much less than the flux quantum. Therefore, we have to divide the device into a large number of thin sections.

Due to the invariance along *x* of all the physical and geometrical parameters, within each section the four envelope functions of monolayer graphene $F_{\beta }^{\vec{\alpha }}\left(\vec{r}\right)$ (corresponding to the sublattices β=A,B and to the Dirac points $\vec{\alpha }=\vec{K},{\vec{K}}^{\prime }$) can be written as the products of a plane wave propagating along *x* and of a transverse component depending only on *y*: $F_{\beta }^{\vec{\alpha }}\left(x,y\right)=e^{i\kappa_{x}x}\Phi_{\beta }^{\vec{\alpha }}\left(y\right)$. In each section, if we introduce the function (defined in the domain $\left[0,2\tilde{W}\right]$)

(1) $$\vec{\varphi }\left(y\right)\;=\;\left\{\begin{array}{cc} \;\;\;e^{-i\tilde{K}y}\left[\begin{array}{c} \;\;\Phi_{A}^{\vec{K}}\left(y\right)\;\;\\ \;\;\Phi_{B}^{\vec{K}}\left(y\right)\;\; \end{array}\right]\; & \;\;y\;\in \;[0,\tilde{W}]\\ \;\;\;e^{i\tilde{K}\left(2\tilde{W}-y\right)}i\left[\begin{array}{c} \;\;\Phi_{A}^{{\vec{K}}^{\prime }}\left(2\tilde{W}-y\right)\;\;\\ \;\;\Phi_{B}^{{\vec{K}}^{\prime }}\left(2\tilde{W}-y\right)\;\; \end{array}\right]\; & \;\;y\;\in \;[\tilde{W},2\tilde{W}]\;, \end{array}\right.$$

the Dirac equation with Dirichlet boundary conditions for the electron wave function is equivalent [57,67] to the following differential system with periodic boundary conditions on the domain $\left[0,2\tilde{W}\right]$:

(2) $$\left\{\begin{array}{cc} \; & \left(\left(\partial_{y}+i\tilde{K}\right)\sigma_{z}+g\left(y\right)I+d\left(y\right)\sigma_{z}+f\left(y\right)\sigma_{x}+q\left(y\right)\sigma_{y}\right)\vec{\varphi }\left(y\right)=-\kappa_{x}\vec{\varphi }\left(y\right)\\ \; & \vec{\varphi }\left(2\tilde{W}\right)=\vec{\varphi }\left(0\right)\;, \end{array}\right.$$

where $\partial_{y}=d/d\;y$; $\tilde{W}$ is the effective width of the ribbon; *e* is the elementary charge; ℏ=h/(2π) is the reduced Planck constant (with *h* the Planck constant); $\sigma_{x}$, $\sigma_{y}$ and $\sigma_{z}$ are the Pauli matrices; *E* is the injection energy; $g\left(y\right)=\left(e/\hbar \right)\;A_{x}(\bar{x},\tilde{W}-|\tilde{W}-y\left|\right)$; $d\left(y\right)=i\;\left(e/\hbar \right)\;\mathrm{sign}\left(\tilde{W}-y\right)A_{y}(\bar{x},\tilde{W}-|\tilde{W}-y\left|\right)$; $f\left(y\right)=[U(\bar{x},\tilde{W}-|\tilde{W}-y|)-E]/\left(v_{F}\hbar \right)$; $q\left(y\right)=-i\;[m(\bar{x},\tilde{W}-|\tilde{W}-y|)v_{F}^{2}]/\left(v_{F}\hbar \right)$; $\bar{x}$ is the abscissa identifying the section; and $\tilde{K}=|\vec{K}\left|-\mathrm{round}\;(\right|\vec{K}\left|\tilde{W}/\pi \right)\pi /\tilde{W}$. Our choice of gauge strongly simplifies the solution of the Dirac Equation (2) in each section. Indeed, with the adopted gauge, the envelope functions $F_{\beta }^{\vec{\alpha }}\left(x,y\right)$ within each section in the presence of magnetic field coincide with those for B=0 multiplied by a Peierls phase factor $\exp[-i\left(e/\hbar \right)A_{y}y]$. Therefore, it is sufficient to perform a numerical solution of the problem (2) in the absence of a magnetic field. This eigenproblem with periodic boundary conditions can be very efficiently treated in the reciprocal domain, thereby obtaining the values of $\kappa_{x}$ and of the Fourier coefficients of $\vec{\varphi }\left(y\right)$, and hence the four envelope functions.

Then, by enforcing the continuity on the two graphene sublattices *A* and *B* of the four envelope functions at the interface between neighboring sections, we have computed the scattering matrix of the transverse region containing this interface [57]. Finally, the scattering matrices of all the regions into which we have divided the device have been composed, thereby obtaining the overall scattering matrix, and, in particular, the transmission matrix *t*. The conductance *G* and the Fano factor *F* (i.e., the ratio of the value of the actual shot noise power spectral density $S_{I}$ to the “full” value 2eI of the shot noise power spectral density that would be expected if charge carriers moved independently, with *I* the average current flowing through the device) have been obtained using the Landauer–Büttiker approach:

(3) $$G=\frac{2e^{2}}{h}\sum_{i}w_{i}\;,\;F=\frac{S_{I}}{2eI}=\frac{\sum_{i}w_{i}\left(1-w_{i}\right)}{\sum_{i}w_{i}}\;,$$

where the $w_{i}$s are the eigenvalues of the matrix $t^{†}t$.

The number of transport modes considered in the calculation was chosen in such a way that a further increase of this number would not alter the transport results within a given accuracy.

At the entrance and at the exit of the considered nanostructure, we have included two regions with low potentials, which emulate the effects of the input and output contacts and guarantee the injection of a sufficient number of propagating modes into the ribbon.

The correctness of the presented approach has been confirmed by performing some tests also with the alternative gauge $\vec{A}=-By\hat{x}$: in this case the Peierls phase technique is not applicable anymore and we have to actually solve Equation (2) with a nonzero magnetic field (again operating in the reciprocal space).

**Figure 2:**
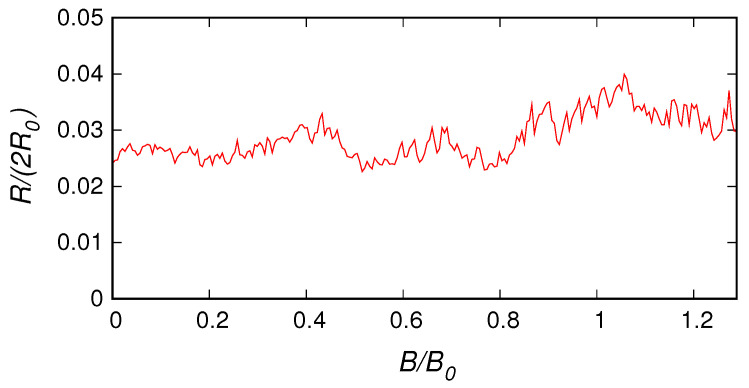
Half of the two-probe resistance (normalized with respect to the resistance quantum $R_{0}=h/\left(2e^{2}\right)$) achieved with our envelope-function-based code for a nanopatterned armchair ribbon with the same size as that studied in reference [52], reported as a function of the orthogonal magnetic field (normalized with respect to $B_{0}=97$ T, the value corresponding to the primary commensurability peak).

We have also used our code to simulate the results of a conductance measurement on a nanopatterned graphene nanoribbon that is available in the literature. In reference [52] a 100 nm × 210 nm zigzag graphene ribbon containing an 8 × 4 square array of antidots (each one with a diameter of 10 nm and a separation of 26 nm) has been simulated using a tight-binding technique in order to analyze the experimental results obtained in reference [49]. Due to the small antidot size and separation (a choice dictated by the computational limits of the tight-binding simulation) and to the large Fermi energy (chosen in such a way as to fall inside the semiclassical regime), in reference [52] large, physically unfeasible values of the magnetic field have been used, in order to explore the commensurability regime. Here, for validation purposes, we repeat a very similar simulation using our envelope function code, considering an armchair graphene ribbon with the same geometry and size as in reference [52], i.e., a 100 nm × 210 nm armchair graphene ribbon containing an 8 × 4 square array of antidots with a diameter of 10 nm and a separation of 26 nm. The Fermi energy is 1.08 eV and we use a mass term of 10 eV. The results of our simulation are reported in Figure 2, with the magnetic field normalized with respect to $B_{0}=97$ T (the value corresponding to the primary commensurability peak) and the resistance normalized with respect to the resistance quantum $R_{0}=h/\left(2e^{2}\right)$. Comparing our numerical result with the data from reference [52] yields good qualitative agreement. The commensurability peaks were properly reproduced, as were the Aharonov–Bohm oscillations, which have a smaller amplitude in the experimental results, due to the presence of dephasing.

## 3. Numerical Results

In order to obtain an aspect ratio among those commonly used in experiments and an overall size yielding a problem of manageable computational complexity, we have considered a 110 nm wide and 200 nm long armchair graphene ribbon (corresponding to a width of 894 dimer lines) containing a set of circular antidots, all with a radius of r= 5.41 nm and located in its central 100 nm long region (see Figure 1). We have analyzed both a regular distribution of antidots, located on a hexagonal lattice with distance between the antidot centers equal to $L_{1}=$ 26.84 nm, and an irregular one, obtained by shifting the center of each antidot (independently along the *x* and *y* directions) by a random quantity uniformly distributed between −4 and 4 nm, in such a way as to simulate the effect of irregularities in the fabrication of the antidots. The effect of the perforations has been introduced by means of a mass term $mv_{F}^{2}$ equal to 1 eV inside the antidots and null outside. We have considered a potential equal to zero in the central 100 nm long region of the sample and equal to −0.2 eV in the input and output leads, in order to simulate the effect of electrical contacts. We have considered two different types of transition between these two potential levels: an abrupt one and a smooth one. The first is step-like: taking x=0 at the beginning of the structure, the potential varies from −0.2 eV to zero at x=20 nm and from zero to −0.2 eV at x=180 nm, to finally end at x=200 nm. The second one starts from −0.2 eV at x=0; from x=0 to x=40 nm rises from −0.2 eV to zero according to the smooth relationship −0.2eV+0.2eV((1+tanh((x−20nm)/(6.67nm)))/2); and finally from x=160 nm to x=200 nm decreases from zero to −0.2 eV according to: −0.2eV+0.2eV((1+tanh((−x+180nm)/(6.67nm)))/2). In our calculations, we had to consider up to 10,000 sections in order to satisfy the constraint on the maximum magnetic flux threading each section.

**Figure 3:**
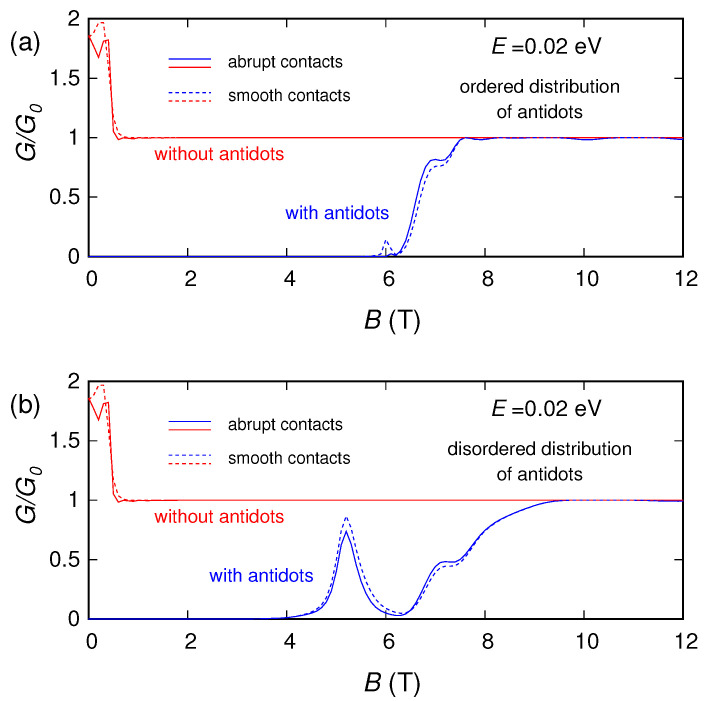
Normalized conductance of the graphene ribbon as a function of the orthogonal magnetic field, obtained for E=0.02 eV in the presence of an ordered (panel (**a**)) and a disordered (panel (**b**)) distribution of antidots, compared with the conductance of the ribbon without antidots.

**Figure 4:**
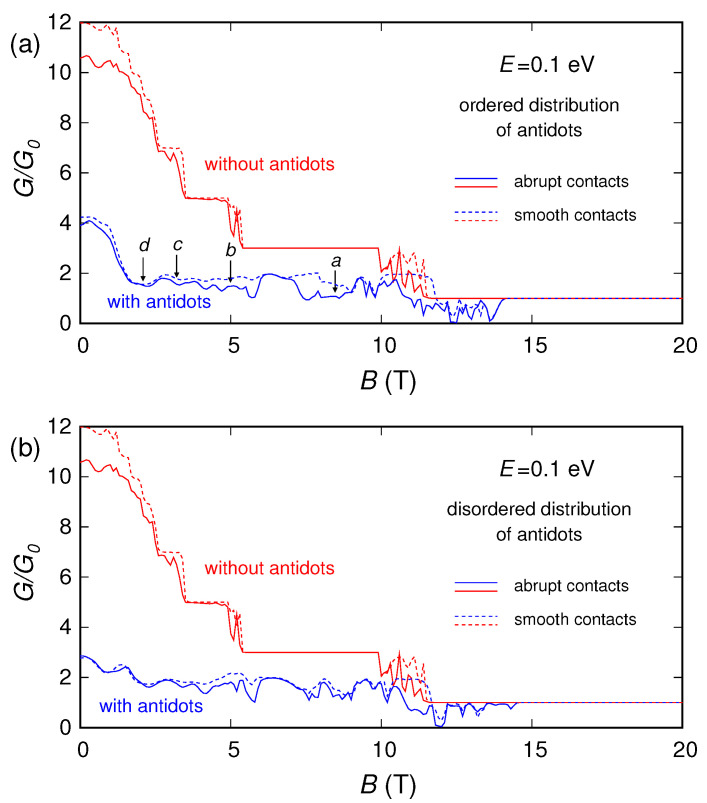
Normalized conductance of the graphene ribbon as a function of the orthogonal magnetic field, obtained for E=0.1 eV in the presence of an ordered (panel (**a**)) and a disordered (panel (**b**)) distribution of antidots, compared with the conductance of the ribbon without antidots.

**Figure 5:**
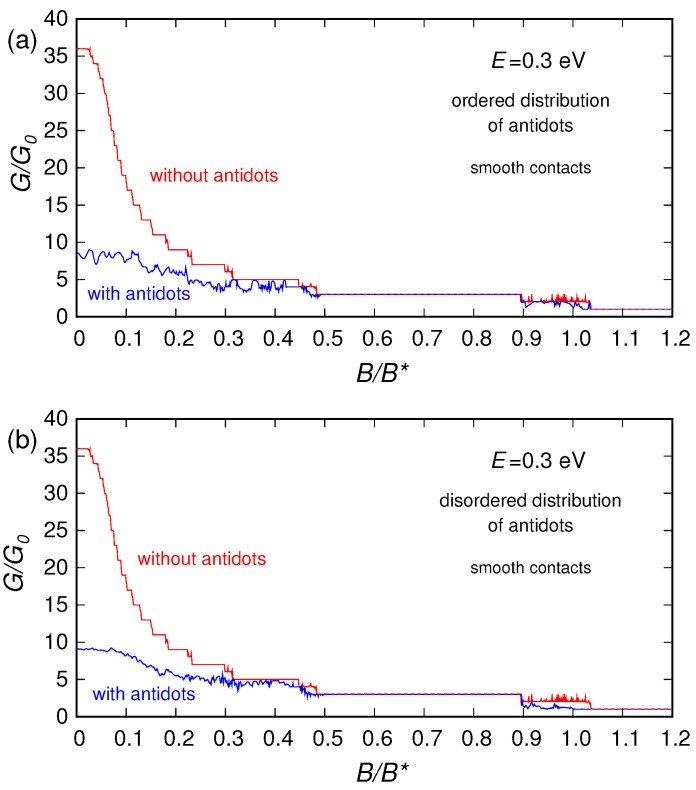
Normalized conductance of the graphene ribbon as a function of the orthogonal magnetic field (normalized with respect to $B^{*}=100$ T), obtained for E=0.3 eV in the presence of an ordered (panel (**a**)) and a disordered (panel (**b**)) distribution of antidots, compared with the conductance of the ribbon without antidots.

In Figure 3, Figure 4 and Figure 5 we report (for three different values of the Fermi energy *E*: 0.02, 0.1 and 0.3 eV) the conductance (normalized with respect to the conductance quantum $G_{0}=2\;e^{2}/h$) that we obtained as a function of the applied magnetic field, for: the pristine ribbon (without the antidots), the ribbon with a regular lattice of antidots and the ribbon with an irregular distribution of antidots. For E=0.02 eV and E=0.1 eV calculations were performed using both a smooth and an abrupt connection between the contacts and the device, obtaining, however, similar behaviors. For E=0.3 eV, instead, we have considered only a smooth connection between the contacts and the device (and in this case, in order to achieve a better understanding of the phenomenon, the calculation was performed up to unrealistically high values of magnetic field).

For no magnetic field, the conductance for E=0.02 eV is zero, while the conductances for E=0.1 eV and E=0.3 eV are different from zero. Indeed, in the absence of magnetic field an energy gap in the order of 0.06 eV is present, generated by the lattice of antidots (the main effect, in this case) [45,46] and by the lateral confinement of the graphene structure [14,33,34]. Since E=0.02 eV falls inside the energy-gap region for B=0 T, for E=0.02 eV we observe a conductance behavior that starts from zero and becomes nonzero only for higher values of the magnetic field. The transition details depend on the specific geometrical distribution of the antidots: in the case of an ordered distribution of antidots (with evenly spaced perforations), the transition is smoother, while in the presence of disorder in the antidot arrangement the transition is more irregular.

**Figure 6:**
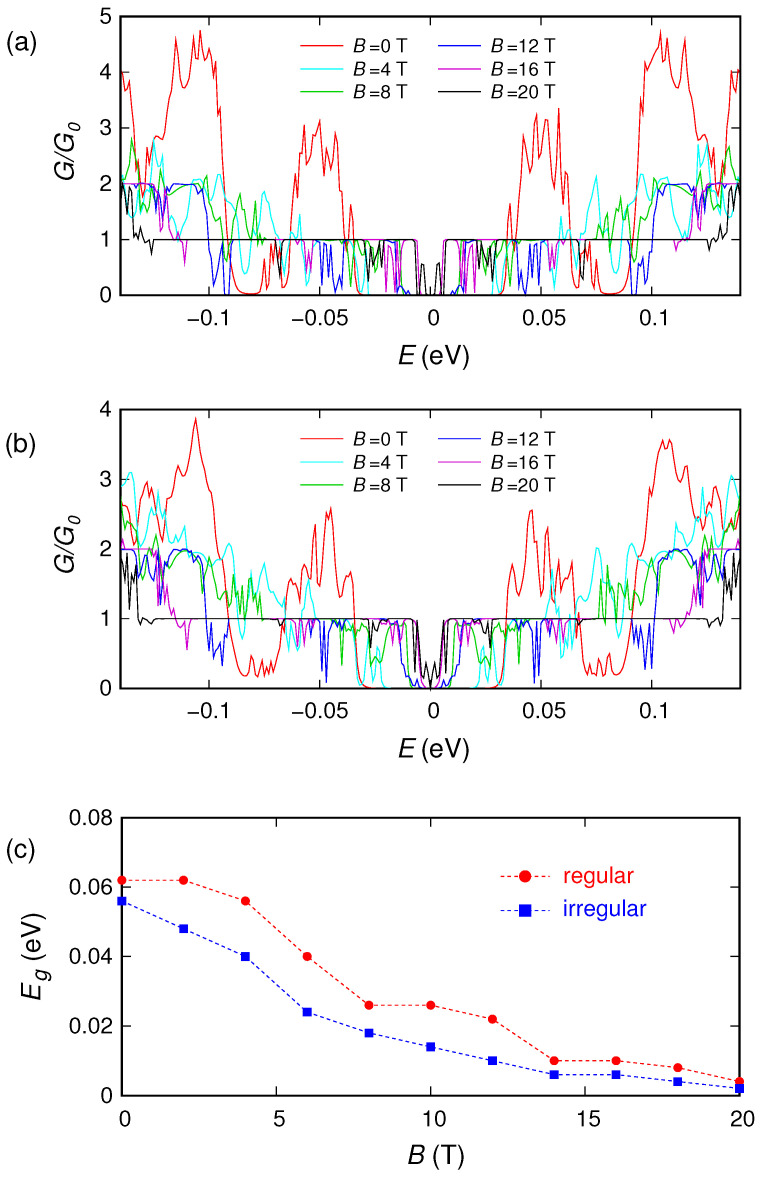
Normalized conductance as a function of the Fermi energy *E* for 6 values of the magnetic field, for the ribbon with an ordered (**a**) and a disordered (**b**) distribution of antidots. In panel (**c**) we report the behavior of the energy gap as a function of the magnetic field for the two different distributions of antidots.

In order to analyze the effect of the magnetic field on the energy gap, in Figure 6 we report the behavior that we have obtained (using a smooth connection between the contacts and the sample) for the conductance as a function of the Fermi energy *E* for several values of the magnetic field, for the cases of ordered and disordered antidots. We see that, starting from an energy gap of the order of 0.06 eV for B=0, by applying an orthogonal magnetic field and progressively increasing its strength, the width of the gap gradually decreases. As mentioned earlier in the introduction, this represents a generalization of what has been previously reported in references [51,62] for unperforated, confined graphene ribbons and for perforated, unconfined graphene, respectively. In Figure 6c we report the behavior of the energy gap (defined as the energy range around zero in which the normalized conductance is less than 0.01) as a function of the magnetic field.

We notice that this agrees with the behavior (reported in Figure 3) of the conductance as a function of the magnetic field for 0.02 eV (an energy smaller than the energy gap in the absence of magnetic field $E_{g}\left(B=0\right)=0.06$ eV). Indeed, for an energy $\tilde{E}$ which falls inside the gap for no magnetic field ($\tilde{E}<E_{g}\left(B=0\right)$), the conductance vs. magnetic field starts from zero, and then becomes nonzero for the value $\tilde{B}$ of the magnetic field such that $E_{g}\left(\tilde{B}\right)=2\tilde{E}$. Actually, in Figure 3 the transition of the conductance takes place for B=6 T, and in Figure 6c we see that $E_{g}\left(6\;T\right)=2\cdot 0.02\;\mathrm{eV}=0.04\;\mathrm{eV}$.

Now let us focus on the values of energy and magnetic field for which the sample has a nonzero conductance. In particular, let us consider the behavior of the conductance as a function of the magnetic field obtained for E=0.1 eV and shown in Figure 4 (an analogous discussion is valid for E=0.3 eV). In the absence of antidots, we notice a clear staircase behavior. The thresholds correspond to the values of *B* for which the considered Fermi energy *E* equals the Landau levels. This can be verified considering, for the sake of simplicity, the Landau levels of unconfined graphene; i.e., $E_{N}^{\pm }=\pm \sqrt{2\hbar v_{F}^{2}eNB}$ (with *N* an integer number). In the absence of confinement, for high values of the magnetic field *B* the electrons can propagate only through the Landau state with N=0, because only the Landau level for N=0 (i.e., $E_{0}=0$) is lower than the injection energy *E*. Therefore, for high values of *B* the conductance is equal to $G_{0}$ (the conductance quantum). By reducing the magnetic field *B*, the Landau levels decrease and therefore the number of propagating modes increases. When *B* reaches the value $B_{1}=9.95$ T, the doubly degenerate Landau level $E_{1}$ equals the injection energy and thus the conductance jumps to $3G_{0}$. Similarly, when the magnetic field becomes lower than 4.98, 3.31 or 2.49 T, the doubly degenerate Landau levels $E_{2}$, $E_{3}$ and $E_{4}$ drop below the injection energy, and the conductances jump to $5G_{0}$, $7G_{0}$ and $9G_{0}$, respectively. It can be observed that also in our case, although actually a confinement is present, the conductance exhibits a behavior as a function of the magnetic field similar to that described in the absence of confinement.

When the antidots are present, the behavior is less regular and several minima and oscillations appear. These features can be explained with the effects studied in GaAs/AlGaAs (gallium arsenide/aluminium gallium arsenide) heterostructures in reference [68] and in graphene in references [48,49,52]. Using a semi-classical approximation, we can assume that the charges move along cyclotron orbits with radius $R_{c}=E/\left(ev_{F}B\right)$, giving rise along the boundaries to edge states crawling in the transport direction [53]. This approximation is better for higher values of *E* (such as 0.1 and 0.3 eV, which correspond to lower Fermi wavelengths), because a semi-classical approximation is accurate if the Fermi wavelength is sufficiently small with respect to the geometrical feature sizes of the device. As a rule of thumb for the validity of the semi-classical approximation, we can compare [52] the Fermi wavelength $\lambda_{F}=hv_{F}/E_{F}$ with the neck width between the antidots $L_{1}-2r$: for 0.1 and 0.3 eV $\lambda_{F}$ is comparable or smaller than the neck width, while for 0.02 eV quantum effects still play a significant role. For the values of magnetic field, and therefore of $R_{c}$, for which the cyclotron orbits encircle an antidot or a group of antidots, the charges, localized around their orbit centers, do not contribute to transport, and this results in conductance dips (commensurability minima). In our hexagonal lattice geometry, when the cyclotron orbit encircles 1, 3, 7 or 12 antidots (see Figure 1a), it gives rise to conductance minima (indicated by arrows in Figure 4a) for B=8.5, 4.9, 3.1 and 2.4 T, respectively (for E=0.1 eV). Quantum effects make the behavior more complex, introducing diffusive scattering, interactions between the different orbits and oscillations (with a period ΔB=(e/h)/A, where *A* is the area of the antidot lattice unit cell) due to the Aharonov–Bohm interference between the components of the wave function scattered by an antidot [49].

**Figure 7:**
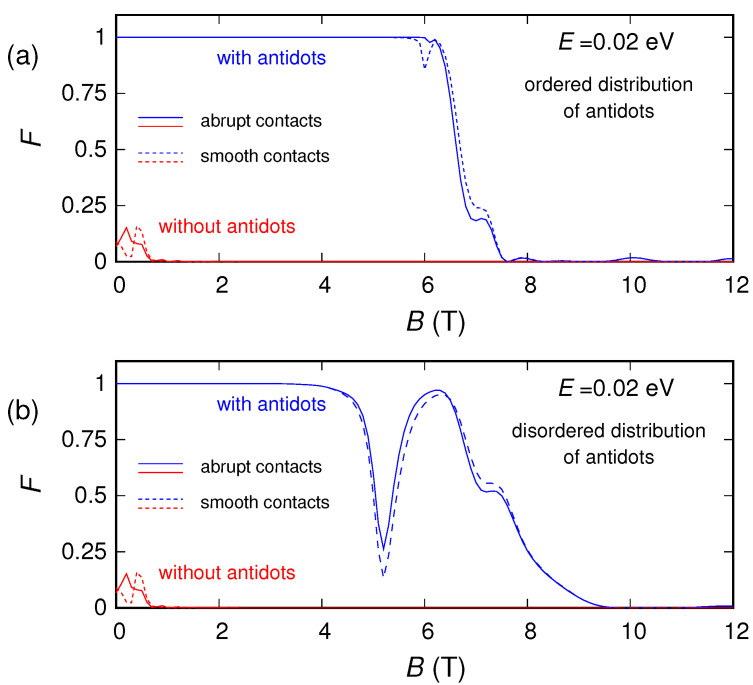
Fano factor of the graphene ribbon as a function of the orthogonal magnetic field, obtained for E=0.02 eV in the presence of an ordered (panel (**a**)) and a disordered (panel (**b**)) distribution of antidots, compared with the Fano factor of the ribbon without antidots.

**Figure 8:**
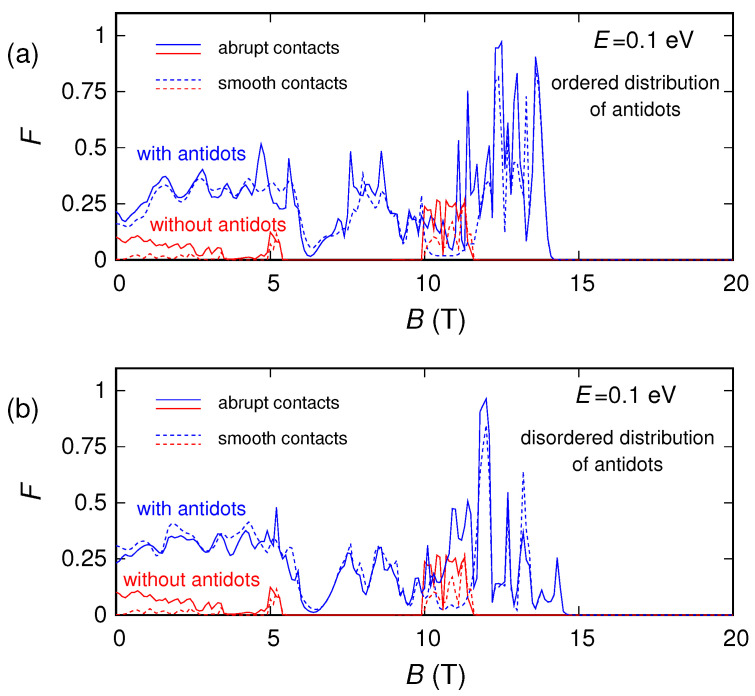
Fano factor of the graphene ribbon as a function of the orthogonal magnetic field, obtained for E=0.1 eV in the presence of an ordered (panel (**a**)) and a disordered (panel (**b**)) distribution of antidots, compared with the Fano factor of the ribbon without antidots.

**Figure 9:**
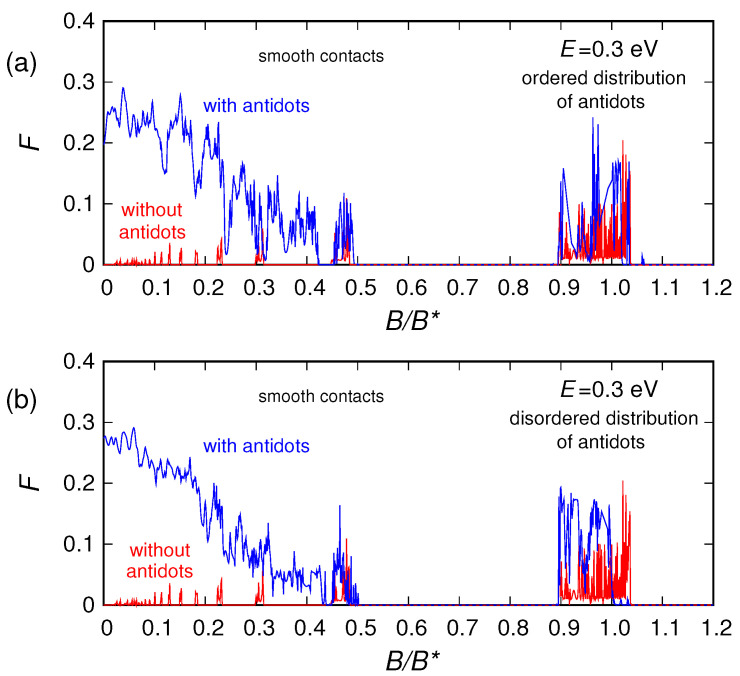
Fano factor of the graphene ribbon as a function of the orthogonal magnetic field (normalized with respect to $B^{*}=100$ T), obtained for E=0.3 eV in the presence of an ordered (panel (**a**)) and a disordered (panel (**b**)) distribution of antidots, compared with the Fano factor of the ribbon without antidots.

Finally, let us look at the conductance and noise behavior for high magnetic fields (which represents the main focus of this work). By examining the behavior of the conductance as a function of the magnetic field *B*, we notice that for large enough *B* the effect of the antidots vanishes. Indeed, for the energies and magnetic fields for which an energy gap is not present, from a semiclassical point of view the motion of the charges can be described in terms of cyclotron orbits. By increasing the magnetic field *B*, the cyclotron radius $R_{c}=E/\left(ev_{F}B\right)$ [53] decreases. When the cyclotron diameter $2R_{c}$ becomes of the order of the average distance $L_{1}-2r$ between adjacent antidots, the charges move along the edges of the structure (Figure 1b) without experiencing any scattering from the antidots, which, therefore, do not affect the transport properties of the device. A similar effect is observed for shot noise, and in particular for the shot noise suppression factor (Fano factor). In Figure 7, Figure 8 and Figure 9 we report the Fano factor as a function of the applied magnetic field, for the same structures and energies considered in Figure 3, Figure 4 and Figure 5. As we can observe, the Fano factor vanishes when the normalized conductance assumes integer values, because in those conditions a number of transport modes (corresponding to the Landau levels lower than the considered Fermi energy) are transmitted with unit probability across the device, while the others do not propagate. We notice that also in this case for a large enough magnetic field the results obtained with and without antidots coincide. In particular, in these conditions, transport becomes ballistic: the edge states crawl along the boundaries without experiencing any scattering due to the antidot lattice. Therefore, each transport mode is either evanescent or totally transmitted; i.e., the transmission probability of each transport mode is either zero or one. As a consequence, no shot noise is present and the Fano factor vanishes, exactly as in the absence of the antidot lattice. This phenomenon is very similar to what we observed in reference [64] for a disordered wire in a GaAs/AlGaAs heterostructure, where the effect of disorder actually disappeared for high values of the magnetic field.

## 4. Conclusions

We have presented an extension of our envelope-function simulator with the inclusion of a mass term (in order to define regions in which particles cannot penetrate) and of the effect of magnetic field, in order to study the transport and noise behavior in an armchair graphene ribbon of a realistic size, with an antidot lattice and an orthogonal magnetic field. We have observed that, as for a graphene sheet, in the case of a confined perforated graphene structure, the energy gap decreases as the magnetic field is increased. Moreover, our results show that, as could be expected from the analogy with ordinary disordered semiconductors, for high values of the magnetic field, the effects of the antidot lattice on the conductance and noise behavior of the structure vanish, as a consequence of the formation of the edge states moving along the boundaries of the structure. In such conditions, transport becomes ballistic and shot noise vanishes. Therefore, such an effect is insensitive to the particular nature of the material. From the point of view of the application of graphene-based devices to digital electronics, nanopatterning with an array of antidots does indeed lead to a gap significantly larger that the one achievable by means of lateral confinement alone; however, the antidots act also as scatterers, thereby lowering the maximum achievable current and therefore the resulting effective mobility. Thus, the antidot density and layout must be chosen carefully, as a result of a trade-off between gap opening and conductance suppression. Our simulation approach and the presented results can be of assistance in establishing such a trade-off, and, in general, in the design and optimization of graphene-based devices and sensors operating in the presence of magnetic fields.
